# Training on an Appetitive (Delay)-Conditioning Task Enhances Oscillatory Waves During Sleep in the Cortical and Amygdalar Network

**DOI:** 10.3389/fnbeh.2018.00260

**Published:** 2018-11-07

**Authors:** Shweta Tripathi, Pankaj Taneja, Sushil K. Jha

**Affiliations:** ^1^Department of Biotechnology, School of Engineering and Technology, Sharda University, Greater Noida, India; ^2^School of Life Sciences, Jawaharlal Nehru University, New Delhi, India

**Keywords:** SWA, learning, NREM sleep, REM sleep, sigma waves, theta waves

## Abstract

Oscillating waves during sleep play an essential role in memory consolidation. The cortical slow wave activity (SWA) and sigma waves during NREM sleep and theta waves during REM sleep increase after a variety of memory tasks including declarative, procedural and associative learning tasks. These oscillatory waves during sleep help to promote neural dialog between circuitries, which possibly plays a causal role in memory consolidation. However, the role of sleep-associated oscillating waves in a complex appetitive-conditioning paradigm is not clear. The parietal cortex and amygdala are involved in the cognitive evaluation of the environmental stimuli, and appetitive conditioning. Here, we have studied the changes in sleep architecture and oscillatory waves during NREM and REM sleep in the parietal cortices and amygdalar-local field potential (A-LFP) after appetitive-conditioning in the rat. We observed that REM sleep increased significantly after appetitive conditioning, which significantly positively correlated with performance on the appetitive-conditioning task. Further, the cortical SWA (0.1–4.5 Hz), and sigma (12–14.25 Hz) waves during NREM sleep, theta (6–9 Hz) waves during REM sleep, the amygdalar SWA (0.1–3.75 Hz) during NREM sleep and theta (6–8.25 Hz) waves during REM sleep significantly increased after appetitive conditioning. Interestingly, the augmented oscillatory waves significantly positively correlated with the performances on the appetitive-conditioning task. Our results suggest that the augmented REM sleep after conditioning may be required for the consolidation of appetitive-conditioned memory. Further, a significant correlation between augmented power in oscillatory waves during sleep and performance suggesting that these waves may be playing a crucial role in the consolidation of appetitive-conditioned memory.

## Introduction

The connection between sleep states and memory processes has been of great interest (Maquet et al., 2003). There are many pieces of evidence to suggest that distinct sleep states are involved in the processing of different types of memory. For example, NREM sleep, sleep spindle and slow waves during NREM sleep increase after fear conditioning, odor-reward association task and skilled reaching task in rodents (Eschenko et al., 2006; Hellman and Abel, 2007; Hanlon et al., 2009; Kumar and Jha, 2012, 2017)and verbal memory retention and motor learning tasks in humans (Walker and Stickgold, 2004; Clemens et al., 2005). Similarly, increase in REM sleep has also been observed after learning a variety of tasks, such as, negative and positive reinforcement task, spatial learning task, avoidance task, classic aversive and appetitive conditioning task in rodents (Smith and Rose, 1997; Walker, 2009; Chowdhury et al., 2011; Rasch and Born, 2013; Tripathi and Jha, 2016) and visual discrimination and procedural learning tasks in humans (Stickgold et al., 2000; Peigneux et al., 2003). These studies suggest that NREM and REM sleep may play a distinctive role in the processing of learning information about different tasks.

Studies suggest that sleep and its parameters play an essential role in the processing of different memories. But its role in complex conditioned-learning paradigms is not clear. Several reports have demonstrated that theta waves (6–9 Hz) during REM sleep, slow wave activity (SWA) (0.1–4 Hz) and sigma waves (11–15 Hz) during NREM sleep may be involved in memory processing (Smith et al., 2004; van der Helm and Walker, 2009 Walker, 2009; Kaestner et al., 2013; Mednick et al., 2013; Rasch and Born, 2013; Hutchison and Rathore, 2015; Headley and Paré, 2017). It has been observed that spindle density increases after learning declarative memory task (Gais et al., 2002), procedural learning task (Fogel and Smith, 2006; Fogel et al., 2007), verbal memory task (Smith, 1985; Clemens et al., 2005), and odor-reward pairing task (Eschenko et al., 2006). In addition, spindles correlated with the hippocampal sharp-wave complexes and reactivated trace memory (Siapas and Wilson, 1998; Sirota and Buzsaki, 2005; Johnson et al., 2010). Further, studies suggest that functional interactions between the hippocampus and cortex during offline states are necessary for long-term stabilization of memory traces. The sharp wave-ripples, SWA, and spindles during sleep help promote the hippocampal-cortical dialog, which seems to play a causal role in the consolidation of the hippocampal-dependent memory (Maingret et al., 2016; Latchoumane et al., 2017). Therefore, it has been reasoned that NREM sleep, which appears with spindles and oscillatory waves, possibly offers the neuronal network a favorable state for the induction of synaptic plasticity, which in turn, helps in memory consolidation (Sejnowski and Destexhe, 2000; Steriade and Timofeev, 2003).

Theta activity, an oscillatory field potential, prominently appears during REM sleep in the hippocampus, amygdala, and neocortex (Brankack et al., 2012; Hutchison and Rathore, 2015). Several reports suggest that theta waves are strongly associated with cognitive and affective functions (Siapas et al., 2005; Sigurdsson et al., 2010). For example, REM sleep as well as theta power increase following avoidance training task (Fogel et al., 2009). Interestingly, oscillations in the hippocampal and amygdalar theta rhythm are synchronized during the retrieval of long-term but not short-term or remote fear memory (Seidenbecher et al., 2003; Narayanan et al., 2007). The coherent theta activity may thus promote memory consolidation (Popa et al., 2010). It has also been observed that attenuating REM sleep theta rhythm selectively after learning by optogenetically silencing the medial septal’s GABAergic neurons induced memory deficit of the hippocampal-dependent tasks (Boyce et al., 2016). These studies suggest that REM sleep theta rhythm may also be playing a causal role in the hippocampal-dependent memory consolidation.

Similar to aversive events, appetitive conditioning is also considered evolutionarily very significant for survival. It remarkably influences the motivational or approach behavior for food differently in conducive and hostile environments; appetitive conditioning has been less studied in animals (Martin-Soelch et al., 2007). Anatomically and functionally, the parietal cortex and central nucleus of amygdala not only play an essential role in attention and cognitive evaluation of the environmental stimuli but also in the learning processes of appetitive conditioning tasks (Dayan et al., 2000; Knapska et al., 2006). Excitotoxic lesion of the central nucleus of amygdala but not the basolateral amygdala induces impairment in the appetitive conditioning task (Parkinson et al., 2000). In addition, total short-term sleep deprivation alters the consolidation of appetitive-conditioned memory (Chowdhury et al., 2011; Tripathi and Jha, 2016). We have also observed that although NREM sleep did not change, REM sleep increases significantly after appetitive conditioning (Tripathi and Jha, 2016). These studies suggest that sleep plays an essential role in the consolidation of appetitive-conditioned memory. It is not known, however, if the oscillatory waves in the cortical and amygdalar networks during NREM and REM sleep play a role in the consolidation of appetitive-conditioned memory. Here, we have studied the changes in sleep architecture and oscillatory waves in different frequency bands in the cortical EEG [SWA (0.1–4.5 Hz), theta (6–9 Hz) and sigma (11–15 Hz)] and the amygdalar-local field potential (A-LFP) [SWA (0.1–4.5 Hz) and theta waves (6–9 Hz)] after appetitive conditioning.

## Materials and Methods

Male Wistar rats (250–300 g) (*n* = 7) were used in this study. Animals were obtained from the university’s Central laboratory of the animal resource (CLAR) and brought to the in-house animal facility for 1 week before the commencement of experiments. Animals were maintained in 12:12 h light:dark cycle (lights on 7:00 AM) in a temperature controlled (23–24°C) environment. Food and water were given *ad libitum*. All procedures and protocols were approved by the Institutional Animal Ethical Committee (IAEC) (IAEC protocol # 09/2007) of Jawaharlal Nehru University, New Delhi, India.

### Surgical Procedures

Animals were surgically prepared for chronic sleep-wakefulness (S-W) recordings. The animal was anesthetized using isoflurane inhalation anesthesia. After anesthetizing the animal, the scalp was shaved, and the head was fixed in the stereotaxic instrument. Skull was exposed by a midline incision on the head skin. Two pairs of small, stainless-steel screw electrodes (length: 2 mm; diameter: 1 mm and head size: 2 mm) were fixed on the frontal (AP: 2 mm; ML: +2 mm and -2 mm, from bregma) and parietal (AP: -2 mm; ML: +2 mm and -2 mm from bregma) bones to record electroencephalogram (EEG) from the frontal and parietal cortices. One screw electrode was fixed laterally to the midline of the nasal bone as a ground electrode. Two flexible insulated wires (except at the tip) were implanted in the neck muscles to record electromyogram (EMG). In addition, one bipolar electrode was implanted in the central nucleus of amygdala (coordinates from the Bregma: AP: -2.5; ML: 4.2; DV: 8.0 mm) to record A-LFP (Paxinos and Watson, 2008). To record SWA and theta activity reliably from the amygdalar region, we have used two 100 μm thick stainless steel wires (California Fine Wire Company, United States). We have kept the length of one the wires 2 mm shorter than other. The two wires were glued together with epoxy glue in such a way that the vertical distance between the two recording-poles, implanted in the brain, was 2 mm away from each other. The free ends of the EEG, EMG, bipolar electrode and ground electrodes were connected to a 9-pin connector, which was cemented onto the skull with dental acrylic. The neck skin was then sutured, and the animal was removed from the stereotaxic instrument. The animal was treated with dexamethasone (1.5 mg/kg) to reduce brain inflammation and nebasulf powder (antibiotic) to control infection post-operatively. Drugs were given for 3–4 days, and the animal was given a week for complete recovery.

### Appetitive-Conditioning

Two distinct paradigms of classical conditioning, trace and delay conditioning, are widely used to study learning and memory. In trace conditioning experiments, the conditioned stimulus (CS) is presented first, and the unconditioned stimulus (US) is presented after the termination of the CS. Thus, both stimuli essentially remain separated by a ‘trace’ interval-time. While, in delay conditioning, the US remains temporally contiguous with the CS, which induces robust learning responses because both the CS and US are presented in close temporal proximity. Further, in classical conditioning, usually either appetitive or aversive stimulus is used as the US. The appetitive stimulus elicits approaching responses, whereas, aversive stimulus elicits avoiding or escaping responses. Also, the trace-conditioned memory requires the hippocampus but not the delay conditioned-memory (Woodruff-Pak and Disterhoft, 2008). The role of sleep-associated oscillatory waves in the hippocampal-dependent task has been widely investigated, but their role in amygdala-dependent memory has not be studied in detail. Hence, we have used the amygdala-dependent appetitive conditioning task in this study.

Animals were trained for appetitive conditioning in a small behavioral conditioning chamber (12” × 12” × 11”) as has been used previously (Tripathi and Jha, 2016). The behavioral conditioning chamber was kept inside the light and sound dampened black box (48” × 24” × 24”) to minimize external disturbances during experiments. Diffuse light of 20 lux was maintained in the experimental chamber during the experiments. The house light (located on the roof of the behavioral chamber) was used as conditioned stimulus (CS), while mango fruit juice (Tropicana Product, PepsiCo India, Gurugram, India) was used as US. The CS and US were presented in a paired fashion to the animal through Graphic State software, (Coulbourn Inc., United States). The house light (used as the CS) was fixed above the juice dispensing unit in behavioral conditioning chamber. The fruit juice (used as the US) was delivered through juice dispensing unit (Coulbourn Inc., United States). The animal had to poke head into the liquid dipper of juice dispensing window on the side wall of the behavioral chamber to obtain juice. The liquid dipper (a cup attached to the lever) of the juice dispensing unit (attached to a computer controlled motor) was automatically filled with 100 μl juice per presentation from the juice-tank placed at the bottom of the dispensing unit. The number of head entries in the liquid dipper to obtain juice (as an outcome measure of learning) was counted by a photo beam sensor (attached on both sidewalls of the juice-dispensing window) and was registered by graphic state software.

**FIGURE 1:**
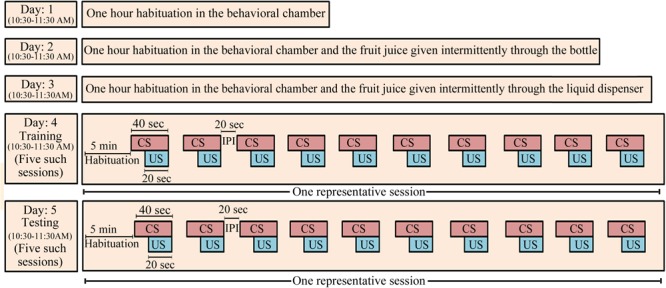
The appetitive-conditioning protocol: Rats were first habituated to the experimental condition on three consecutive days daily for 1 h (10:30–11:30 AM). On day 1, the animals were habituated to the conditioning chamber for 1 h. On day 2, the animals were again habituated to the conditioning chamber for 1 h, but mango fruit juice was given manually through a small bottle intermittently during habituation. It was done so that the animals develop a taste for the juice. On day 3, the mango juice was given to the animal for about 15–20 times through the liquid dispenser of the juice-dispensing unit during the habituation. The experimenter manually operated the juice-dispensing unit and guided the animal toward the juice-dispensing window. Training and testing of the appetitive-conditioned task were performed on subsequent days. House light in the behavior chamber was used as a conditioned stimulus, and fruit juice was used as unconditioned stimulus. CS, conditioned stimulus; US, unconditioned stimulus; IPI, inter-presentation interval.

We followed the same protocol as reported earlier (Tripathi and Jha, 2016) (Figure 1). On day 1, the animal was first habituated in the behavioral conditioning chamber for 1 h (10:30–11:30 AM). On day 2, the animal was again kept in the behavioral chamber for 1 h and was exposed to the fruit juice (mango juice, Tropicana Product, PepsiCo India, Gurugram, India). The fruit juice was given four to five times through a nozzle fitted glass bottle. This was done to familiarize the animal with the taste and aroma of the juice and to develop a desire to obtain the fruit juice. On day 3, juice was presented to the animal, through the liquid dipper of the juice-dispensing unit (Coulbourn Inc., United States). The experimenter manually lifted the lever of the juice-filled cup of the juice-dispensing unit and guided the animal toward the juice-dispensing window for about 15–20 times to familiarize the animal about the juice delivery route in the dispensing window. With this, the animal exhibited an eagerness to obtain fruit juice and started approaching the juice delivery window to obtain the juice on its own after this small training. Animals that did not approach the window quickly were guided again on the next day or else they were considered outliers. After habituation and exposure to the fruit juice, the animal was conditioned on day 4 between 10:30 AM and 11:30 AM. The protocol of Graphic State software (Coulbourn Inc., United States) was written in such a way that after 5 min of initial habituation period, it delivered the CS (house light) for 40 s, which co-terminated with 20 s of US presentation followed by a 20 s inter-presentation interval (Figure 1). The paired CS–US was presented over a time period of 1 h in five sessions. In each session, 10 paired CS–US were presented with 2 min inter-session interval. Next day (on day 5), the animal was tested for conditioned memory using the same protocol as was used during the training. The animal was kept in the conditioning chamber and was allowed to explore the chamber for 5 min. During the post-conditioning session, the CS and US were presented in a similar way as were presented during the training period. The number of head entries into the juice-dispensing window was registered in the computer, as an outcome measure.

### Polysomnographic Recordings

After 1 h habituation in the behavior conditioning chamber (10:30 AM to 11:30 AM), the animal was habituated in the sleep recording chamber for 6 h (11:30 AM to 5:30 PM) on day 1. During the habituation, the animal was tethered to the recording cable and polysomnographic recording setup through a commutator. The recording chamber was illuminated with 20–30 lux light. After habituation, S-W was recorded for four consecutive days [day 2: baseline day (exposure to fruit juice through the bottle), day 3: hand-poke training day (juice delivery through liquid dipper), day 4: appetitive-conditioned training day, and day 5: appetitive conditioned testing day] for 6 h between 11:30 AM and 5:30 PM.

EEGs from the frontal and parietal cortices were recorded in two separate channels (EEG recordings from the parietal cortex was only used for power spectrum analysis). The bipolar A-LFP and EMG from the neck muscles were recorded in an individual channel. The signals were recorded strictly using the same recording cable and electrode combinations in the same animals across days. Electrophysiological signals were filtered and amplified using 15LT Bipolar Portable Physiodata Amplifier System (Astro-Med, United States). EEGs and A-LFP signals were processed with a high-pass filter of 0.1 Hz and a low-pass filter of 40 Hz, while EMG signal was processed with a high-pass filter of 10 Hz and a low-pass filter of 90 Hz digitized at 100 Hz sampling rate. Recordings were acquired in a computer using Spike2 software (Cambridge Electronic Design, Cambridge, United Kingdom) and were saved for offline analysis. Representative polygraphic traces of EEG, A-LFP, and EMG during Wakefulness, NREM, and REM sleep along with cortical and A-LFP power spectral profile are shown in Supplementary Figure S1.

### Data Analysis

#### Analysis of Appetitive-Conditioned Task

Changes in the number of head entries (an outcome measure of learning) on appetitive-conditioned training and testing days were compared statistically. A total number of head entries in the juice-dispensing window during all five sessions in all animals were registered into the computer through the Graphic state software. Overall changes in the total number of head entries during the CS–US presentation on the appetitive-conditioned training and testing days were examined by means of box-and-whisker plots. The changes in the total number of head entries on the training and testing day were compared statistically using non-parametric Wilcoxon Signed Rank test. In addition, the total number of head entries across five sessions were compared using one-way repeated measures analysis of variance (RM-ANOVA) followed by Bonferroni *post hoc* test.

#### Analysis of S-W

Offline, polysomnographic Spike2 records were converted into the European data format and were analyzed by Somnologica Science software (Medcare Flaga, Iceland). Computerized polysomnographic records were manually scored using 4-s epochs for 6 h period from 11:30 AM to 5:30 PM employing the standard criteria for rats. Low voltage and high-frequency EEG waves associated with increased motor activity were analyzed as wake; high voltage, low-frequency EEG waves and decreased motor activity were analyzed as NREM sleep, and low voltage, high-frequency EEG waves with a prominent theta peak and nuchal muscle atonia were analyzed as REM sleep.

The total mean percent time spent in wakefulness, NREM, and REM sleep on the baseline, hand-poking, training and testing days were calculated and statistically compared with baseline days (one-way RM-ANOVA followed by Bonferroni *post hoc* test). The changes in percent REM sleep amount (out of 6 h total recording time) were also examined by means of box-and-whisker plots and compared statistically using Wilcoxon Signed Rank test. The changes in average REM sleep amount at every 2 h on the baseline, hand-poking, training and testing days were also calculated and statistically compared with baseline days (one-way RM-ANOVA followed by Bonferroni *post hoc* test). Further, the changes in REM sleep amount at the two-hourly window (0–2 h, 2–4 h, and 4–6 h) was correlated with the number of head entries using Pearson correlation test.

#### Power Spectral Analysis of Cortical EEG and A-LFP

Learning-dependent changes in the power of artifact-free cortical EEG and A-LFP during different vigilant state (Wake, NREM, and REM sleep states) were calculated using spectral analysis in Somnologica science software. Fourier transformed EEG, and A-LFP recorded from the parietal cortex and amygdala, respectively, were normalized to the mean power of individual EEG and A-LFP frequencies range, respectively, across all Wake, NREM, and REM sleep episodes (EEG recorded from the frontal cortex was not used for power spectral analysis). We first generated power-spectral values across all frequencies (0.1–25 Hz at 0.75 Hz bin size) for each Wake, NREM, and REM sleep episode for the entire recording period [using Somnologica Science software (Medcare Flaga, Iceland)]. The power values between 0.1 and 25 Hz across the individual vigilant state were then averaged within the animal (without detrending) to obtain a single normalized value. The single normalized value for Wake, NREM, and REM sleep (within animal) was used to divide the original power spectrum frequencies for each state. This normalization corrects the differences in absolute EEG power between recordings in different animals. We then averaged EEG spectral power in each vigilance state into SWA (0.1–4.5 Hz), theta (6–9 Hz) and sigma (11–15 Hz) frequency bands. We have used the same normalization method earlier to generate average spectral profiles of EEGs in NREM and REM sleep state before and 6 h after sleep deprivation (Jha et al., 2006; Aton et al., 2009). Every two-hourly average EEG’s and A-LFP’s spectral profiles were calculated with percent baseline to determine the relative changes in different EEG frequency bands. The changes were compared statistically (one-way RM ANOVA followed by Bonferroni *post hoc* test). We have also applied Pearson correlation test to correlate the performance (the number of head entries) with the percent increase in the power of EEG’s and A-LFP’s oscillatory waves during the two-hourly window.

### Histology

**FIGURE 2:**
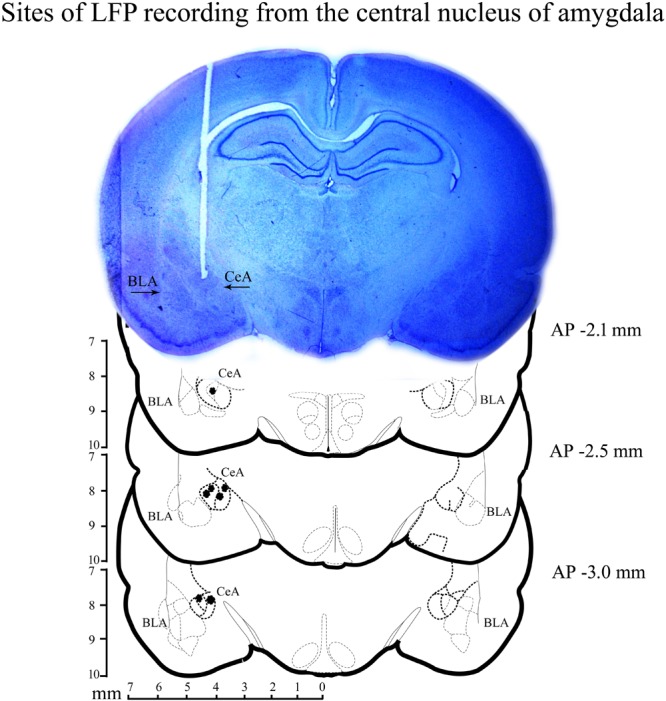
A histological microphotograph of 40-μm cresyl violet stained coronal section of the rat brain and reconstruction diagram showing the sites of local field-potential (LFP) recording from the central nucleus of amygdala. Filled circles (•) denote the sites of recording.

After the completion of experiments, animals were anesthetized with an overdose of ketamine (80 mg/kg) and xylazine (10 mg/kg) cocktail. They were then perfused transcardially with 0.9% saline followed by 10% formaldehyde. The brain was preserved in 10% formalin and later placed in 30% sucrose solution. Coronal sections were cut and stained with cresyl violet. The sites of bipolar electrode placement were identified under a microscope. A representative microphotograph and reconstruction diagram exhibiting the sites of recordings within the central nucleus of amygdala are shown in Figure 2.

## Results

### The Number of Head Entries in the Juice Dispensing Chamber on the Appetitive-Conditioned Training and Testing Days

**FIGURE 3:**
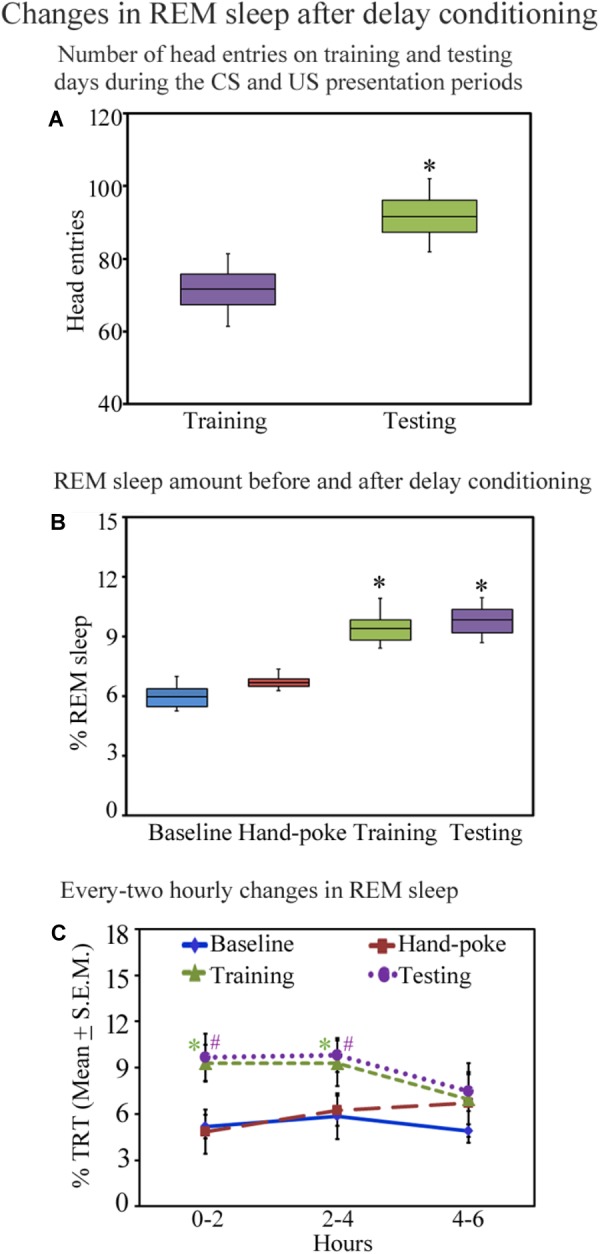
The number of head entries during the appetitive-conditioned training and testing periods and percent REM sleep amount on different days before and after appetitive conditioning are depicted in the box and whisker plots as median, interquartile range, minimum, and maximum values. **(A)** The number of head entries was significantly more on the testing day compared to the training day (*p* < 0.05; Wilcoxon Signed Rank test). **(B)** The percent REM sleep amount was also more on the appetitive-conditioned training (*p* < 0.05) and testing days (*p* < 0.05) (Wilcoxon Signed Rank test). **(C)** The percent REM sleep amount at every two-hourly periods on the baseline, hand-poking, appetitive-conditioned training, and testing days. The percent REM sleep amount significantly increased [*p* < 0.05; *F*(3,27) = 6.78] during 0–2 h (Bonferroni corrected *p* < 0.05) and 2–4 h (Bonferroni corrected *p* < 0.05) periods on the training day compared to the baseline day (one-way RM ANOVA). REM sleep also significantly increased on the testing day during 0–2 h (Bonferroni corrected *p* < 0.05) and 2–4 h (Bonferroni corrected *p* < 0.05) periods [*p* < 0.05; *F*(3,27) = 5.82] (compared to the baseline day) (one-way RM ANOVA). ^∗^ and # denote *p* < 0.05. TRT, total recording time, SEM, standard error of mean.

All animals trained for the appetitive-conditioned task performed well during the testing. The number of head entries during the CS–US paired presentation periods on the testing day was more (median = 91.68; interquartile range = 8.8) (*p* < 0.05; *z* = 2.36) (Wilcoxon Signed Rank test) than the training day (median = 71.76; interquartile range = 8.5) (Figure 3A). On the training day, the number of head entries during every five sessions was comparable. On the testing day, however, *post hoc* comparison demonstrate a significant change in the numbers of head entries in every session [*p* < 0.001; *F*(13,69) = 48.42] [the number of head entries increased in session-1 by 35.24% (*p* < 0.001), session-II by 40.18% (*p* < 0.001), in session-III by 29.30% (*p* < 0.001), in session-IV by 25% (*p* < 0.001) in session-V by 21% (*p* < 0.001) compared to the training day] (Supplementary Figure S2). These results suggest that the animals have appropriately learned the appetitive-conditioned task.

### Changes in Sleep Architecture on the Appetitive-Conditioned Training and Testing Days

Sleep architecture changed after appetitive conditioning on the training and testing days. The total amount of wakefulness and NREM sleep did not change (Supplementary Figure S3), but interestingly, REM sleep significantly increased on the training and testing days [*p* < 0.001, *F*(3,27) = 11.17] (one-way RM-ANOVA) compared to the baseline day (Supplementary Figure S3). The median value and interquartile range of percent REM sleep amount on the baseline day were 5.95 and 0.88; hand-poke training day were 6.68 and 0.38. On the appetitive-conditioned training day, percent REM sleep amount was more (median = 9.38; interquartile range = 1.02) (*p* < 0.05; *z* = 2.52 compared to the baseline day) (Wilcoxon Signed Rank test). Similarly, on the testing day, percent REM sleep amount was also more than the baseline day (median = 9.83; interquartile range = 1.16) (*p* < 0.05; *z* = 2.52 compared to the baseline day) (Wilcoxon Signed Rank test) (Figure 3B). In the two-hourly analysis, we observed that REM sleep amount was significantly high during 0–2 h (*p* < 0.05) and 2–4 h (*p* < 0.05) periods on the training day [*p* < 0.05; *F*(3,27) = 6.78] (compared to the baseline day) (Bonferroni-adjusted *p*-value on one-way RM ANOVA). REM sleep also significantly increased on the testing day during 0–2 h (*p* < 0.05) and 2–4 h (*p* < 0.05) periods [*p* < 0.05; *F*(3,27) = 5.82] (compared to the baseline day) (Bonferroni-adjusted *p*-value on one-way RM ANOVA) (Figure 3C). The changes in REM sleep amount during the 4–6 h time period on the training and testing days were, however, statistically not significant (Figure 3C). These results demonstrate that appetitive-conditioned learning influences REM sleep during the early phase of memory consolidation.

### Correlation Between Performance (Head Entries) and REM Sleep Amount on the Training and Testing Days

**FIGURE 4:**
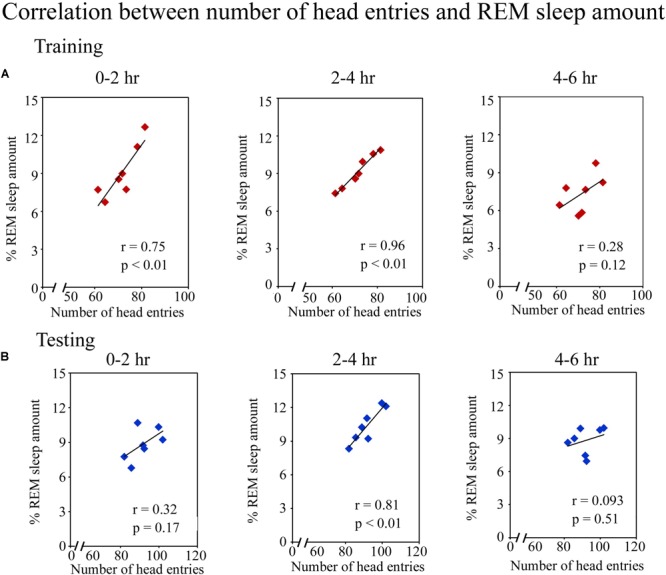
Pearson correlation between performance and percent REM sleep amount. **(A)** The number of head entries significantly positively correlated with the REM sleep amount at 0–2 h (*r* = 0.75; *p* < 0.01) and 2–4 h (*r* = 0.96; *p* < 0.01) periods on the training day. **(B)** It also significantly correlated on the testing day, but only during the 2–4 h (*r* = 0.81; *p* < 0.01) period.

Further, we observed that an early phase of memory consolidation, REM sleep amount significantly correlated with performance (Figure 4). On the training day, the number of head entries positively correlated with the REM sleep amount at 0–2 h (*r* = 0.75; *p* < 0.01) and 2–4 h (*r* = 0.96; *p* < 0.01) time periods. However, it did not significantly correlate at 4–6 h of (*r* = 0.28; *p* = 0.12) (Figure 4A). On the testing day, the number head entries positively significantly correlated with the REM sleep amount only during 2–4 h (*r* = 0.81; *p* < 0.01). It was, however, not significant at 0–2 h (*r* = 0.32; *p* = 0.17) and 4–6 h (*r* = 0.09; *p* = 0.51) (Figure 4B) time periods. Although, number of head entries significantly positively correlated with REM sleep amount at certain time windows on the training and testing days, NREM sleep and wake amount did not significantly correlate with the performance at any time period on either day. These results thus suggest that REM sleep may be essentially required during the early phase of memory consolidation.

### Changes in EEG SWA and Sigma Waves in the Cortical Circuitries During NREM Sleep and EEG Theta Waves During REM Sleep on the Appetitive-Conditioned Training and Testing Days

**FIGURE 5:**
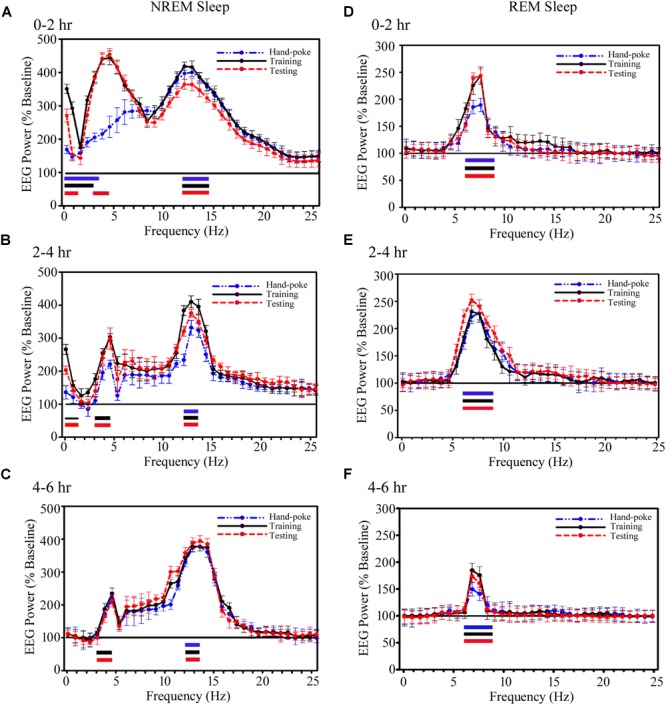
The cortical EEG’s SWA and sigma waves (spindles) recorded from the parietal cortices during NREM sleep and theta waves during REM sleep significantly increased on the training and testing days. **(A)** During NREM sleep, SWA significantly increased in 0.1–3.75 Hz range on the hand-poke training day, 0.1–3.0 Hz range on the training and 0.1–1.5 Hz range and in 3–4.5 Hz range on the testing days during the 0–2 h period. EEG power in sigma range (12–14.25 Hz) during NREM sleep also significantly increased on the hand-poke training, appetitive-conditioned training and testing days during 0–2 h compared to the baseline day. **(B)** SWA significantly increased in 0.1–1.5 Hz and 3–4.5 Hz range during 2–4 h after appetitive-conditioned training and testing. Sigma waves also remained significantly augmented in 12–13.5 Hz during 2–4 h after hand-poke training and appetitive conditioned training and testing. **(C)** Interestingly, SWA remained significantly increased in the high-frequency range (3.0–4.5 Hz) during 4–6 h after appetitive conditioned training and testing. The power of EEGs sigma waves remained consistently augmented in 12–13.5 Hz frequency range during the 4–6 h period after hand-poke training and appetitive-conditioned training and testing. Theta wave (6–9 Hz) during REM sleep remained significantly increased during **(D)** 0–2 h period, **(E)** 2–4 h period, and **(F)** 4–6 h period after hand-poke training and appetitive-conditioned training and testing. Three different colored bars above abscissa: Blue colored bar (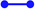) represents significant changes on hand-poke training; black colored bar (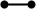) represents significant changes on appetitive-conditioned training, and red colored bar (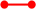) represents significant changes on the appetitive-conditioned testing day compared to the baseline day. The thickness of the colored bars above abscissa: Thick bar (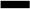) represents Bonferroni corrected *p* < 0.001; thin bar (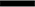) represents Bonferroni corrected *p* < 0.01.

Although, the total amount of NREM sleep did not change, interestingly, the cortical EEG’s SWA and sigma waves during NREM sleep, and theta waves during REM sleep significantly increased on the training and testing days (Figure 5). The power of SWA and theta waves did not change during wakefulness on either day. During NREM sleep, the power of SWA in 0.1–3.75 Hz range significantly increased by 68% on the hand-poke training day (*p* < 0.001), by 245% in 0.1–3.0 Hz range on the training day (*p* < 0.001) and by 165% in 0.1–1.5 Hz (*p* < 0.001) range and by 375% in 3–4.5 Hz (*p* < 0.001) range on the testing day during 0–2 h time period as compared to baseline day (Figure 5A). Whereas, the changes in SWA wave during 2–4 and 4–6 h after hand-poke training were not statistically significant (Figures 5B,C). Interestingly, on the appetitive-conditioned training day, SWA significantly increased by 145% in 0.1–1.5 Hz range (*p* < 0.01) and 205% in 3–4.5 Hz range (*p* < 0.001) during 2–4 h after training. On the appetitive-conditioned testing day, SWA significantly increased by 100% in 0.1–1.5 Hz range (*p* < 0.001) and 187% in 3–4.5 Hz range (*p* < 0.001) during the 2–4 h time period (Figure 5B). Similarly, SWA also significantly increased by 135% in high-frequency range only (3.0–4.5 Hz) during 4–6 h after training (*p* < 0.001) and 120% after testing (*p* < 0.001) (Figure 5C). One-way RM-ANOVA comparison between hand poke vs. appetitive-conditioned training days and hand poke vs. appetitive-conditioned testing days revealed no significant changes in SWA at any time points.

Further, EEG power in the sigma range (12–14.25 Hz) during NREM sleep also significantly increased on the hand-poke training, appetitive-conditioned training and testing days (Figure 5). It increased by 195% (*p* < 0.001) on a hand-poke training day, 350% (*p* < 0.001) on an appetitive-conditioned training day and 360% (*p* < 0.001) on appetitive-conditioned testing days during 0–2 h compared to the baseline day (Figure 5A). The power of EEGs sigma waves remained consistently augmented in 12–13.5 Hz frequency range during 2–4 and 4–6 h time periods by 220% (*p* < 0.001), and 265% (*p* < 0.001) on hand-poke training day, 305% (*p* < 0.001) and 267% (*p* < 0.001) on the appetitive-conditioned training day, 280% (*p* < 0.001) and 200% (*p* < 0.001) on appetitive-conditioned testing day, respectively, compared to baseline day (Figures 5B,C). Also, one-way RM-ANOVA demonstrated that the changes in sigma waves during the training and testing days were statistically not significant from the hand poking day in any two-hourly windows.

Similarly, power of REM sleep theta waves (6–9 Hz) during 0–2 h also significantly increased by 80% (*p* < 0.001) on the hand-poke training, 142% (*p* < 0.001) on appetitive-conditioned training and 148% (*p* < 0.001) on appetitive-conditioned testing days compared to the baseline day (Figure 5D). Theta waves consistently remained elevated during 2–4 and 4–6 h time points by 130% (*p* < 0.001) and 50% (*p* < 0.001) on hand-poke training days, 142% (*p* < 0.001) and 95% (*p* < 0.001) on appetitive-conditioned training days, 151% (*p* < 0.001) and 78% (*p* < 0.001) on appetitive-conditioned testing days compared to baseline days (Figures 5E,F). The changes in the power of other oscillatory waves did not change during Wake, NREM or REM sleep. One-way RM-ANOVA comparison between hand poke vs. appetitive-conditioned training days and hand poke vs. appetitive-conditioned testing days revealed no significant changes in theta waves at any time points. These results show that appetitive-conditioning influences cortical SWA, theta and sigma waves only during sleep.

### Changes in the Power of SWA and Theta Waves in the Amygdalar Circuitries During NREM and REM Sleep on the Appetitive-Conditioned Training and Testing Days

**FIGURE 6:**
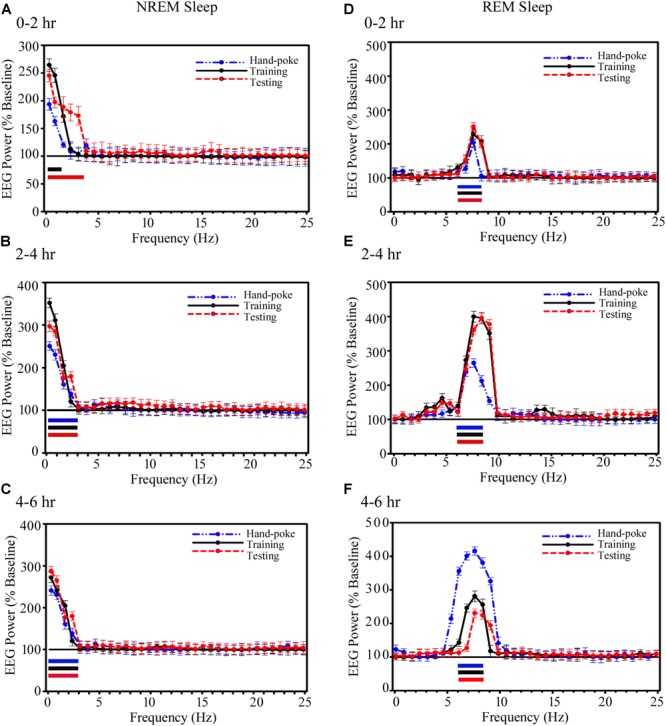
Changes in the amygdalar SWA (0.1–4.5 Hz) during NREM sleep and theta waves (6–8.25 Hz) during REM sleep after hand-poke training, appetitive-conditioned training, and testing. SWA significantly increased during **(A)** 0–2 h **(B)** 2–4 h and **(C)** 4–6 h after hand-poke training, appetitive-conditioned training, and testing. Similarly, theta waves also significantly change during **(D)** 0–2 h **(E)** 2–4 h and **(F)** 4–6 h after hand-poke training, appetitive-conditioned training, and testing. Three different colored bars above abscissa: Blue colored bar (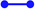) represent significant changes on hand-poke training; black colored bar (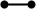) represents significant changes on appetitive-conditioned training, and red colored bar (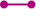) represents significant changes on the appetitive-conditioned testing day compared to the baseline day. The thickness of the colored bars above abscissa: Thick bar (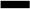) represents Bonferroni corrected *p* < 0.001; thin bar (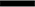) represents Bonferroni corrected *p* < 0.01.

The power in SWA during NREM sleep and theta waves during REM sleep also significantly increased in the A-LFP on the training and testing days (Figure 6). During NREM sleep, the power in EEG SWA (0.1–1.5 Hz) significantly increased by 165% (*p* < 0.001) during 0–2 h on the appetitive-conditioned training day and 145% (*p* < 0.001) in 0.1–3.75 Hz range on the appetitive-conditioned testing day (Figure 6A) as compared to the baseline day. SWA increased by 98% during 0–2 h after hand-poke training, but it was statistically not significant (Figure 6A). Further, the power of SWA (0.1–3 Hz) during NREM sleep significantly increased during 2–4 and 4–6 h by 145% (*p* < 0.001) and 135% (*p* < 0.001) on hand-poke training day, by 250% (*p* < 0.001) and 175% (*p* < 0.001) on appetitive-conditioned training day, by 200% (*p* < 0.001) and 185% (*p* < 0.001) on appetitive-conditioned testing day, compared to the baseline day (Figures 6B,C). The one-way ANOVA revealed no significant changes in SWA’s power between conditions (hand-poke vs. training; hand-poke vs. testing, and training vs. testing).

Similar to the changes in the power of SWA, REM sleep theta waves (6–8.25 Hz) during 0–2 h also significantly increased by 100% (*p* < 0.001) on the hand poke training day, 125% (*p* < 0.001) on appetitive-conditioned training day and 130% (*p* < 0.001) on appetitive-conditioned testing (Figure 6D) day compared to the baseline day. The power in theta waves remain consistently elevated during 2–4 and 4–6 h time points by 165% (*p* < 0.001) and 300% (*p* < 0.001) on hand poke training day, 300% (*p* < 0.001) and 195% (*p* < 0.001) on the appetitive-conditioned training day, 303% (*p* < 0.001) and 140% (*p* < 0.001) on the appetitive-conditioned testing day compared to the baseline day (Figures 6E,F). The changes in A-LFP theta power during 0–2 h on hand-poking, training and testing days were comparable. However, on hand-poking training day, A-LFP’s theta waves mean power was 33.75% (*p* < 0.01) less compared to the appetitive-conditioned training and 34.24% (*p* < 0.01) less compared to the appetitive-conditioned testing days during 2–4 h window. Surprisingly, theta power during 4–6 h time point on the hand-poking day was 35.60% (*p* = 0.19) and 66.67% (*p* < 0.05) more compared to the training and testing days, respectively. The changes in EEG power in other frequency ranges were also statistically not significant during Wake, NREM or REM sleep. These results demonstrate that the appetitive-conditioning also influences oscillatory waves in the amygdalar circuitries.

### Correlation Between Performance (Head Entries) and Power of EEG’s SWA and Sigma Waves During NREM Sleep and Theta Waves During REM Sleep on the Training and Testing Days

**FIGURE 7:**
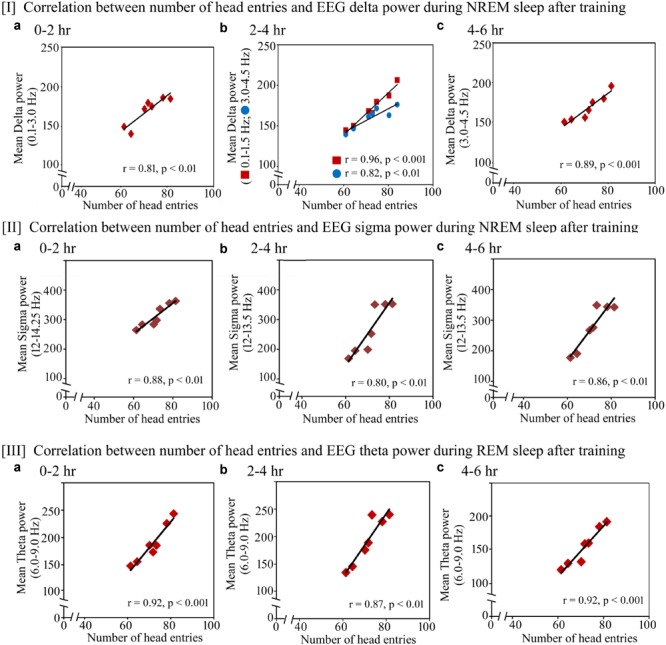
Pearson correlation between performance and percent change in cortical oscillatory waves during NREM and REM sleep during the training day. On the training day, **(I)** the number of head entries significantly positively correlated with the increased power of SWA in the range of 0.1–3 Hz (*r* = 0.81; *p* < 0.01) at 0–2 h **(a)**; 0.1–1.5 Hz (*r* = 0.96; *p* < 0.001) and 3–4.5 Hz (*r* = 0.82; *p* < 0.01) at 2–4 h **(b)**; and 3–4.5 Hz (*r* = 0.89; *p* < 0.001) at 4–6 h **(c)**; **(II)** the increased power in sigma waves during NREM sleep also significantly positively correlated with performance in the range of 12–14.25 Hz at 0–2 h (*r* = 0.88; *p* < 0.01) **(a)** and in the range of 12–13.5 Hz at 2–4 h (*r* = 0.80; *p* < 0.01) and 4–6 h (*r* = 0.86; *p* < 0.01) **(b,c)**; **(III)** theta power during REM sleep in the range of 6–9 Hz also significantly positively correlated with performance during 0–2 h (*r* = 0.92; *p* < 0.001), 2–4 h (*r* = 0.87; *p* < 0.01) and 4–6 h (*r* = 0.92; *p* < 0.001) **(a–c)**.

**FIGURE 8:**
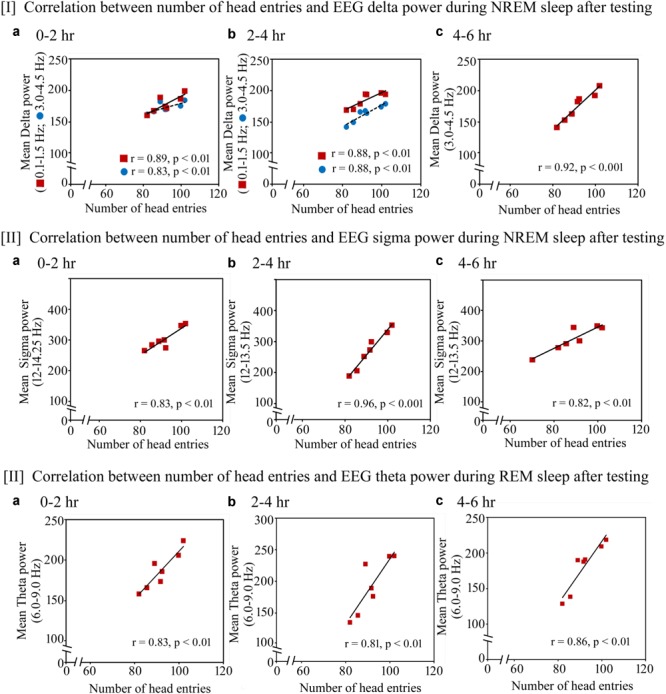
Pearson correlation between performance and percent change in cortical oscillatory waves during NREM and REM sleep during the testing day. On the testing day, **(I)** the number of head entries positively significantly correlated with the increased power of SWA waves in the range of 0.1–1.5 Hz (*r* = 0.89; *p* < 0.01) and 3–4.5 Hz (*r* = 0.83; *p* < 0.01) at 0–2 h **(a)**; 0.1–1.5 Hz (*r* = 0.88; *p* < 0.01) and 3–4.5 Hz (*r* = 0.88; *p* < 0.01) at 2–4 h **(b)** and 3–4.5 Hz (*r* = 0.92; *p* < 0.001) at 4–6 h **(c)**; **(II)** the power in sigma range during NREM sleep also significantly positively correlated with performance in the range of 12–14.25 Hz at 0–2 h (*r* = 0.83; *p* < 0.01) **(a)** and in the range of 12–13.5 Hz at 2–4 h (*r* = 0.96; *p* < 0.001) and 4–6 h (*r* = 0.82; *p* < 0.01) **(b,c)**; **(III)** theta power (6–9 Hz) during REM sleep also significantly positively correlated with performance during 0–2 h (*r* = 0.83; *p* < 0.01), 2–4 h (*r* = 0.81; *p* < 0.01) and 4–6 h (*r* = 0.86; *p* < 0.01) **(a–c)**.

Further, we observed that the performance significantly positively correlated with increased power in SWA and sigma waves during NREM sleep and theta waves during REM sleep on the training and testing days (Figures 7, 8). On the training day, the number of head entries significantly positively correlated with the increased power of SWA in the range of 0.1–3 Hz (*r* = 0.81; *p* < 0.01) at 0–2 h (Figure 7I a); 0.1–1.5 Hz (*r* = 0.96; *p* < 0.001) and 3–4.5 Hz (*r* = 0.82; *p* < 0.01) at 2–4 h (Figure 7I b) and 3–4.5 Hz (*r* = 0.89; *p* < 0.001) at 4–6 h (Figure 7I c) on the training day. Similarly, the increased power in sigma waves during NREM sleep also significantly positively correlated with performance in the range of 12–14.25 Hz at 0–2 h (*r* = 0.88; *p* < 0.01) (Figure 7II a) and in the range of 12–13.5 Hz at 2–4 h (*r* = 0.80; *p* < 0.01) and 4–6 h (*r* = 0.86; *p* < 0.01) (Figures 7II b,c). The increased theta power during REM sleep in the range of 6–9 Hz also significantly positively correlated with performance during 0–2 h (*r* = 0.92; *p* < 0.001), 2–4 h (*r* = 0.87; *p* < 0.01) and 4–6 h (*r* = 0.92; *p* < 0.001) (Figures 7III a–c).

The similar changes in EEG SWA, sigma waves during NREM sleep and theta waves during REM sleep were also observed on the testing day (Figure 8). The number of head entries positively significantly correlated with the increased power of SWA waves in the range of 0.1–1.5 Hz (*r* = 0.89; *p* < 0.01) and 3–4.5 Hz (*r* = 0.83; *p* < 0.01) at 0–2 h (Figure 8I a); 0.1–1.5 Hz (*r* = 0.88; *p* < 0.01) and 3–4.5 Hz (*r* = 0.88; *p* < 0.01) at 2–4 h (Figure 8I b) and 3–4.5 Hz (*r* = 0.92; *p* < 0.001) at 4–6 h (Figure 8I c) on the testing day. Similarly, the increased power in sigma range during NREM sleep also significantly positively correlated with performance in the range of 12–14.25 Hz at 0–2 h (*r* = 0.83; *p* < 0.01) (Figure 8II a) and in the range of 12–13.5 Hz at 2–4 h (*r* = 0.96; *p* < 0.001) and 4–6 h (*r* = 0.82; *p* < 0.01) (Figures 8II b,c). The increased theta power (6–9 Hz) during REM sleep also significantly positively correlated with performance during 0–2 h (*r* = 0.83; *p* < 0.01), 2–4 h (*r* = 0.81; *p* < 0.01) and 4–6 h (*r* = 0.86; *p* < 0.01) (Figures 8III a–c).

### Correlation Between Performance (Head Entries) and Power of A-LFP’s SWA During NREM Sleep and Theta Waves During REM Sleep on the Training and Testing Days

**FIGURE 9:**
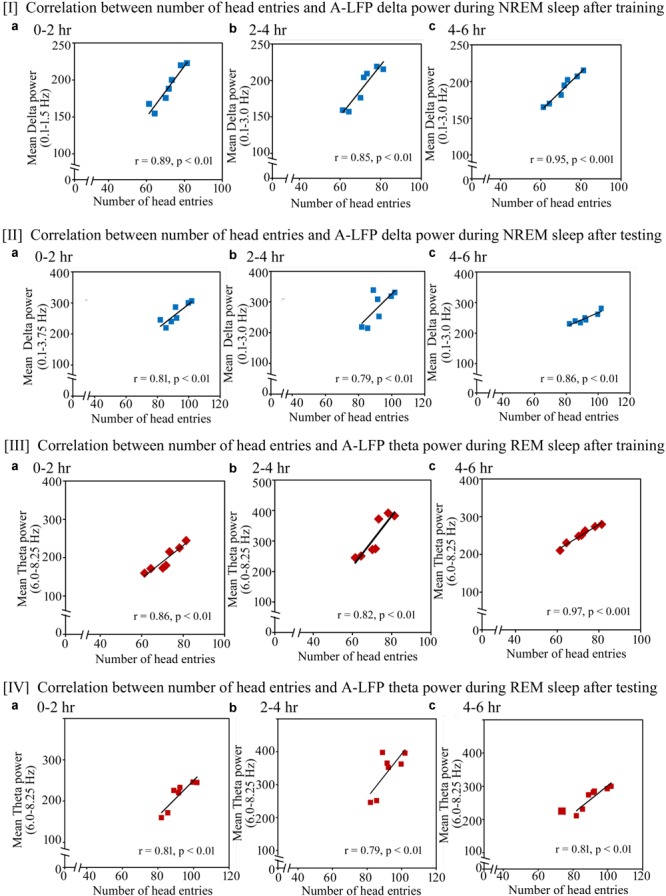
Pearson correlation between performance and percent change in A-LFP oscillatory waves during NREM and REM sleep during the training and testing day. **(I)** On the appetitive-conditioned training day, the number of head entries significantly positively correlated with the increased power of SWA **(a)** in the range of 0.1–1.5 Hz (*r* = 0.89; *p* < 0.01) at 0–2 h, **(b)** in the range of 0.1–3 Hz at 2–4 h (*r* = 0.85; *p* < 0.01), and **(c)** in the range of 0.1–3 Hz at 4–6 h (*r* = 0.95; *p* < 0.001). **(II)** The number of head entries also positively significantly correlated with the increased power of SWA in the range of **(a)** 0.1–3.75 Hz (*r* = 0.81; *p* < 0.01) at 0–2 h; **(b)** 0.1–3 Hz (*r* = 0.79; *p* < 0.01) at 2–4 h; and **(c)** 0.1–3 Hz (*r* = 0.86; *p* < 0.01) at 4–6 h during NREM sleep on the testing day. **(III)** The increased theta power (6–8.25 Hz) during REM sleep also significantly positively correlated with performance during **(a)** 0–2 h (*r* = 0.86; *p* < 0.01), **(b)** 2–4 h (*r* = 0.82; *p* < 0.01), and **(c)** 4–6 h (*r* = 0.97; *p* < 0.001). **(IV)** The increased theta power (6–8.25 Hz) during REM sleep also significantly positively correlated with performance during **(a)** 0–2 h (*r* = 0.81; *p* < 0.01), **(b)** 2–4 h (*r* = 0.79; *p* < 0.01), and **(c)** 4–6 h (*r* = 0.81; *p* < 0.01). A-LFP, amygdalar local filed potential.

Similar to the changes in the cortical EEG’s SWA and theta waves, we found that the performance also significantly positively correlated with increased power in A-LFP’s SWA waves during NREM sleep and theta waves during REM sleep on the training and testing days (Figure 9). On the training day, the number of head entries significantly positively correlated with the increased power of SWA in the range of 0.1–1.5 Hz (*r* = 0.89; *p* < 0.01) at 0–2 h (Figure 9I a); in the range of 0.1–3 Hz at 2–4 h (*r* = 0.85; *p* < 0.01) and at 4–6 h (*r* = 0.95; *p* < 0.001) (Figures 9I b,c) on the training day. The increased theta power (6–8.25 Hz) during REM sleep also significantly positively correlated with performance during 0–2 h (*r* = 0.86; *p* < 0.01), 2–4 h (*r* = 0.82; *p* < 0.01) and 4–6 h (*r* = 0.97; *p* < 0.001) (Figures 9III a–c).

On the testing day, the power in A-LFP’s SWA during NREM sleep and theta waves during REM sleep also significantly correlated with performance (Figure 9). The number of head entries positively significantly correlated with the increased power of SWA in the range of 0.1–3.75 Hz (*r* = 0.81; *p* < 0.01) at 0–2 h (Figure 9II a); 0.1–3 Hz (*r* = 0.79; *p* < 0.01) at 2–4 h (Figure 9II b) and 0.1–3 Hz (*r* = 0.86; *p* < 0.01) at 4–6 h (Figure 9II c) during NREM sleep on the testing day. The increased theta power (6–8.25 Hz) during REM sleep also significantly positively correlated with performance during 0–2 h (*r* = 0.81; *p* < 0.01), 2–4 h (*r* = 0.79; *p* < 0.01) and 4–6 h (*r* = 0.81; *p* < 0.01) (Figures 9IV a–c).

## Discussion

Our findings demonstrate that REM sleep remained significantly increased up to 4 h after appetitive-conditioned training and testing. NREM sleep, however, did not change. The increased REM sleep amount after appetitive-conditioned training significantly positively correlated with the number of head entries. It suggests that increased REM sleep may essentially be required for the consolidation of appetitive-conditioned memory. Further, we observed that SWA and sigma waves during NREM sleep, and theta waves during REM sleep significantly increased in the cortical brain areas after learning the appetitive conditioning task. In addition, we found a corresponding significant increase in SWA and theta waves during NREM and REM sleep in the amygdalar region after appetitive conditioning. Interestingly, the significantly augmented oscillatory waves (SWA, Sigma and theta waves) in the cortical as well as amygdalar circuitries positively correlated with the performances. These results show that the cortical as well as A-LFP’s SWA, sigma and theta waves may be playing an important role in the consolidation of appetitive delay-conditioned memory. These results suggest that appetitive conditioning may require REM sleep for the consolidation of conditioned memory. Our results also suggest that appetitive conditioning task influences sleep-associated EEG’s oscillatory waves such as SWA, theta and sigma waves.

The endogenous slow wave oscillations in SWA range may play a crucial role in the sleep-dependent memory consolidation. A local increase of SWA during sleep has been observed explicitly in learning-associated brain areas, and such augmentation in SWA significantly correlated with improved performance (Huber et al., 2004). On the other hand, SWA significantly decreased during sleep in learning associated brain regions, if the learning was impaired (Huber et al., 2006). Interestingly, EEG SWA also increases during learning while asleep (Arzi et al., 2012). Using partial-reinforcement trace conditioning paradigm, pleasant and unpleasant odors paired with different tones were presented during sleep. It was found that (a) sleeping subjects learned novel associations between tones and odors and (b) most importantly, it was observed that power of SWA and sigma waves significantly increased at a post-tone period during sleep after learning (Arzi et al., 2012). We have also found similar changes in SWA and sigma waves during NREM sleep after learning the appetitive task. These results demonstrate that learning may influence different cortical waves during sleep.

Why learning needs an augmented cortical waves in the SWA, theta, and sigma range, is not precisely known. Interestingly, it has been found that the enhanced cortical oscillations of SWA and spindle-like potential by either magnetic stimulation or pharmacologic interventions after learning help potentiate memory consolidation. Using intermittent transcranial direct-current stimulation (0.75 Hz) technique, Marshall et al., 2006 have found an increase in the EEG power in the slow oscillation band (<1 Hz) during the stimulation-free intervals. Interestingly, the increase was associated with enhanced memory retention (Marshall et al., 2006). On the other hand, it has also been found that some drugs such as gaboxadol and tiagabine induce restorative sleep along with a robust increase in SWA during NREM sleep (Walsh et al., 2006, 2008). Interestingly, compared with the control group, the drug-treated groups exhibited better performances in the psychomotor vigilance test, and Wisconsin card-sorting task after sleep restriction (Walsh et al., 2006, 2008). These further suggest that slow-wave oscillation in SWA range during NREM sleep may be one of the causal factors essentially required for memory consolidation.

A successful memory encoding also accompanies an increase in theta power (Berry and Thompson, 1978; Lega et al., 2012; Hutchison and Rathore, 2015). The hippocampal theta oscillation is involved in the formation of episodic memories, emotional memories, fear memories (Popa et al., 2010; Lega et al., 2012; Hutchison and Rathore, 2015). It has been found that theta activity is synchronized in the amygdala-hippocampal network during the tone presentation after fear conditioning (Seidenbecher et al., 2003), and interestingly, such activity has an affirmative role in the consolidation of fear memories (Pape et al., 2005). On the other hand, if theta oscillation is interrupted/eliminated either through pharmacological interventions or disruption through the medial septal nucleus lesioning, memory consolidation was impaired (Winson, 1978; Givens and Olton, 1995; Boyce et al., 2016). Also, animals learn faster if they exhibit EEG hippocampal waves predominantly in theta range prior to learning compared to the animals exhibiting waves in higher frequencies (8–22 Hz) range (Seager et al., 2002). All these reports suggest that theta waves may be playing a causal role in memory processing.

In our study, we found that cortical sigma waves, cortical and amygdalar SWA waves during NREM sleep and cortical and amygdalar theta waves during REM sleep increased significantly after learning. The slow, as well as fast oscillatory waves, have a role in orchestrating neuronal plasticity and neuronal connections not only during sleep in adult but during development as well (Lindemann et al., 2016; Del Rio-Bermudez et al., 2017; Kurth et al., 2017; Del Rio-Bermudez and Blumberg, 2018). The slow and fast oscillatory waves remain synchronized over a large part of the cortex during NREM sleep, and such long-range network synchronization possibly provides a temporal window for processing and strengthening memory (Aton et al., 2009; Chauvette et al., 2012). Theta waves also exhibit a synchronized pattern in the amygdalo-cortical circuitries, which play a crucial role in potentiating tone induced fear memory (Popa et al., 2010). It is known that tone induced fear memory is amygdala-dependent (Kumar and Jha, 2012, 2017). The central nucleus of amygdala is also involved in attention and reward (Easton and Gaffan, 2000; Parkinson et al., 2000; Tripathi and Jha, 2016) and is one of the loci, where the association between cues with emotional responses takes place (LeDoux, 2000). Although, we have recorded the A-LFP from the central nucleus of amygdala in all seven animals (the sites of recordings were histologically verified) (Figure 2), but the two poles of the bipolar electrode were 2 mm apart from each other. It is likely that the recorded oscillatory activity may not entirely be from the CeA, but also from the adjoining amygdalar areas. There is evidence suggesting that the amygdalar neurons produce theta wave activity during emotional arousal and various rhythmic activities during sleep (Paré and Collins, 2000; Paré et al., 2002). Further, our findings that SWA and theta waves are augmented after learning in the amygdala suggest that it could be involved in the consolidation of appetitive-conditioned memory.

We observed the changes in SWA, theta and sigma waves on not only training and testing days, but also on the hand-poke training day. On the hand-poking day, we trained the animals to obtain juice through a window of the liquid dispenser, and the animals have learned about the juice-dispensing site. It was the part of pre-conditioning protocol and was done only to familiarize the animals with the juice delivery route. SWA and sigma waves significantly increased on this day as well. But, interestingly, we observed the changes in the cortical SWA waves in low-frequency ranges during the initial 0–2 h period. It, however, did not significantly change in subsequent periods. Whereas, on the appetitive-conditioned training and testing days, the SWA waves significantly changed in low-frequency during the 0–2 h and 2–4 h time periods and high-frequency range during 2–4 h and 4–6 h time periods. In the A-LFP recording, SWA waves did not change significantly during the 0–2 h period after the hand-poke training, but it significantly increased after appetitive-conditioned training and testing. The memory related to the location of the juice dispenser unit within the conditioning chamber could be the part of the episodic memory system, whereas the acquired information regarding appetitive-conditioning is part of implicit memory. The change in the oscillatory waves in different frequency range could be attributed to either (a) the nature of memory (explicit/implicit memory) or (b) different memory load on the hand-poke training and appetitive-conditioned training days. It has been reported that an increase in the power of 3-Hz slow oscillatory waves predicts the successful encoding of episodic-memory (Lega et al., 2012). Further, it has also been reported that different memory load may influence oscillatory waves in different frequency ranges (van Vugt et al., 2010). It requires more in-depth study to ascertain if different memory types can influence EEG oscillatory waves in the different frequency range.

## Conclusion

In conclusion, our results suggest that different memory types influence EEG oscillatory waves in different frequency range during the early and late phase of memory consolidation. Our findings demonstrate that (i) REM sleep increased significantly after appetitive conditioning, (ii) it significantly positively correlated with performances, (iii) the cortical SWA, theta and sigma waves significantly increased after appetitive conditioning, and (iv) the amygdalar SWA and theta waves also significantly increased after appetitive conditioning. We could not ascertain the distinctive role of all the outcomes in memory consolidation. It would be interesting to know, however, if any one specific outcome or all of the above changes together may play a causative role in memory consolidation. We, also, could not determine if SWA, theta, and sigma waves all show synchronous activity at some particular temporal window in the amygdalo-cortical network after learning. The precise role of these outcomes can be investigated in the future. Nevertheless, our study shows that appetitive conditioning influence EEG’s oscillatory waves in larger cortical as well as amygdalar circuitries.

## Author Contributions

ST performed the experiments, and generated and analyzed the data. SJ conceived the idea and designed the work, analyzed the data and finalized the manuscript. PT helped in manuscript preparation.

## Conflict of Interest Statement

The authors declare that the research was conducted in the absence of any commercial or financial relationships that could be construed as a potential conflict of interest.

## Supplementary Material

FIGURE S1 Polysomnographic traces along with power spectral profile of cortical EEG and A-LFP during wakefulness, NREM, and REM sleep from a single rat. A-LFP, amygdalar local filed potential.

FIGURE S2 The number of head entries during every five sessions on training and testing days. The number of head entries on the training day in each session was comparable. It, however, significantly increased in each session during testing [*p* < 0.001; *F*(13,69) = 48.42] (one-way RM ANOVA) compared to the training day. ^∗∗∗^denotes Bonferroni corrected *p* < 0.001.

FIGURE S3 The percent sleep-wake amount on the baseline, hand-poke training, appetitive-conditioned training and testing days. **(A)** % wakefulness and **(B)** % NREM sleep were comparable in the baseline, hand-poke training and testing days. However, **(C)** % REM sleep significantly increased on appetitive-conditioned training and testing days [*p* < 0.001; *F*(3,27) = 11.17] (one-way RM ANOVA) compared to baseline day. ^∗∗∗^denotes Bonferroni corrected *p* < 0.001.
