# Supplementary figures and images for: Training on an Appetitive (Delay)-Conditioning Task Enhances Oscillatory Waves During Sleep in the Cortical and Amygdalar Network
Source: Front Behav Neurosci. 2018 Nov 7;12:260. doi: 10.3389/fnbeh.2018.00260 (PMC6234907; doi:10.3389/fnbeh.2018.00260)

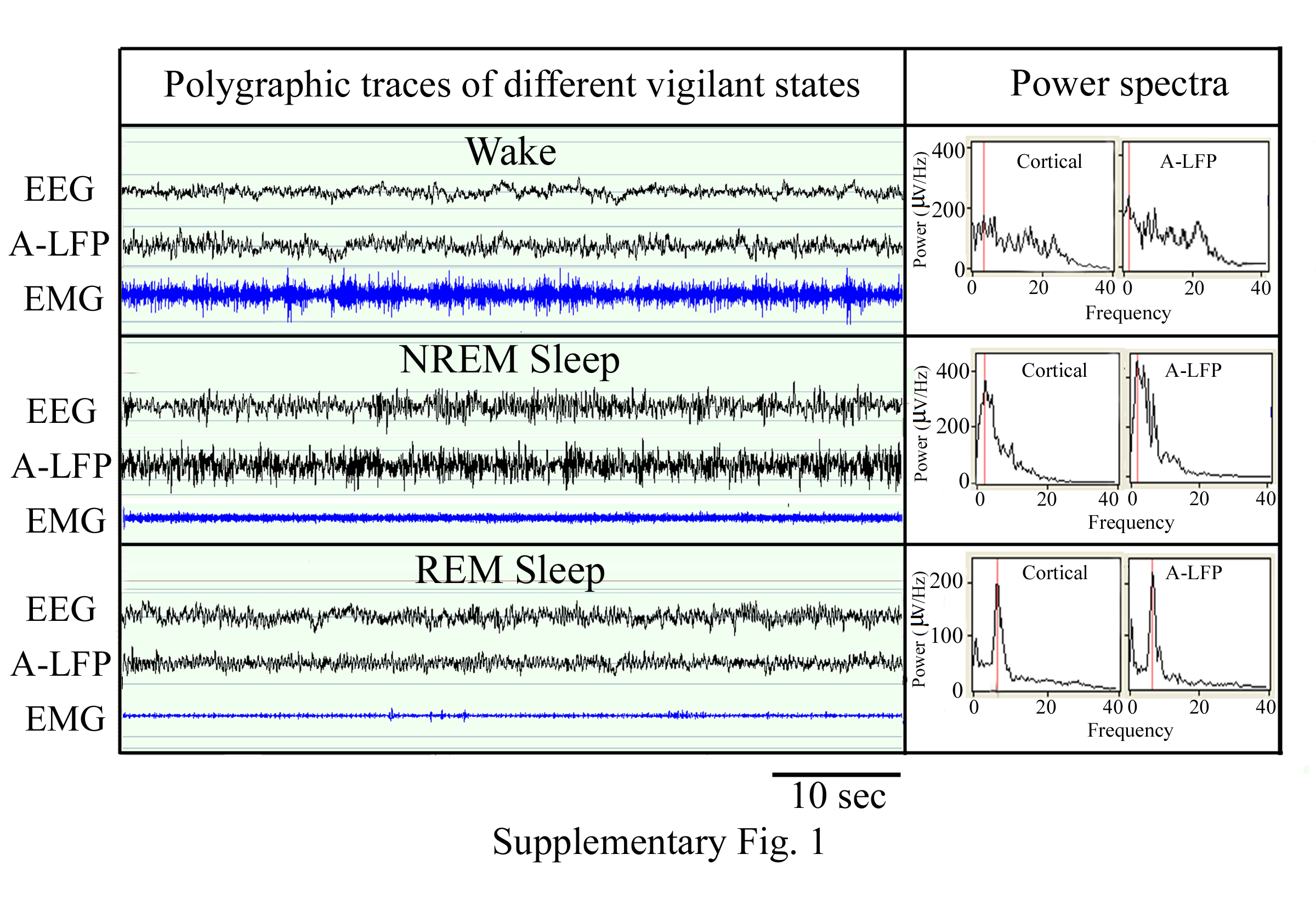

Supplement: FIGURE S1 — Polysomnographic traces along with power spectral profile of cortical EEG and A-LFP during wakefulness, NREM, and REM sleep from a single rat. A-LFP, amygdalar local filed potential. [file Image_1.TIF]

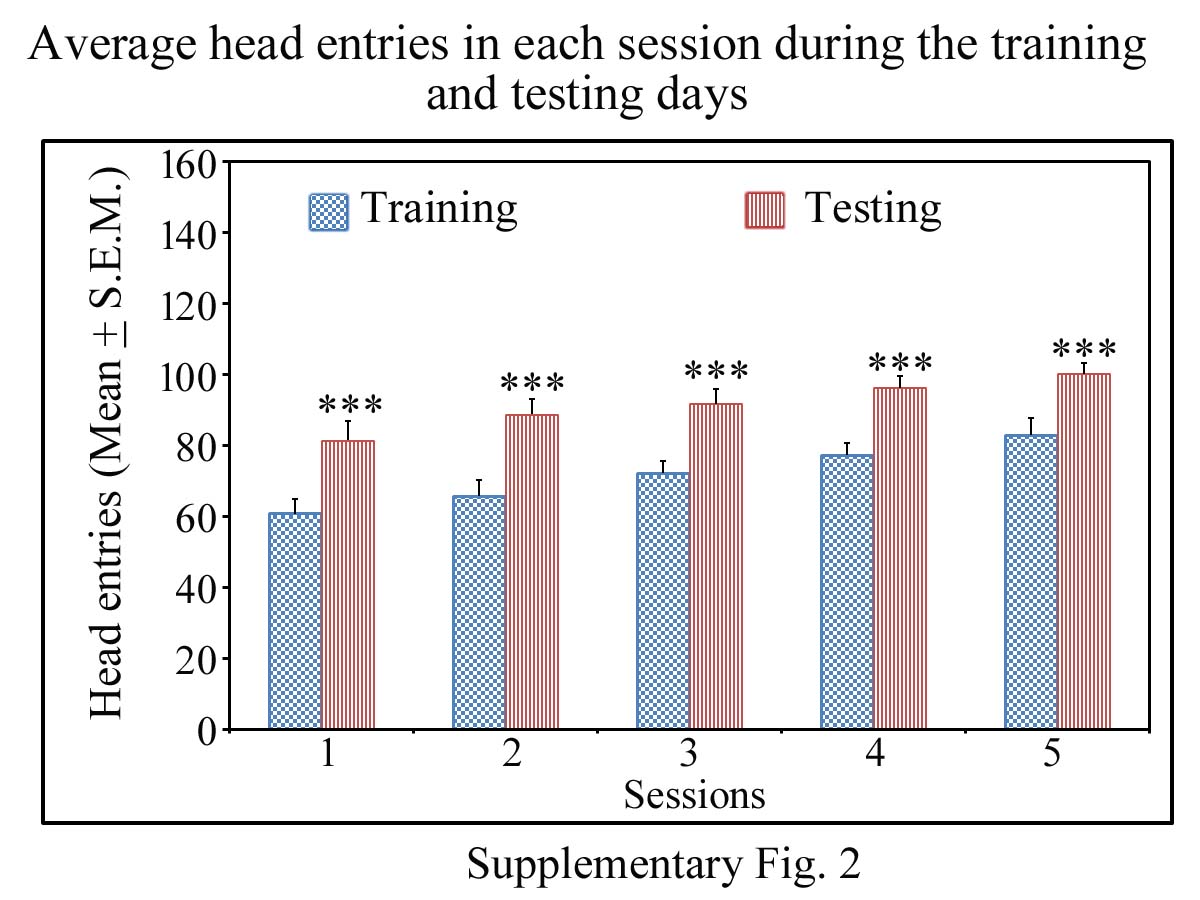

Supplement: FIGURE S2 — The number of head entries during every five sessions on training and testing days. The number of head entries on the training day in each session was comparable. It, however, significantly increased in each session during testing [p < 0.001; F(13,69) = 48.42] (one-way RM ANOVA) compared to the training day. ∗∗∗denotes Bonferroni corrected p < 0.001. [file Image_2.JPEG]

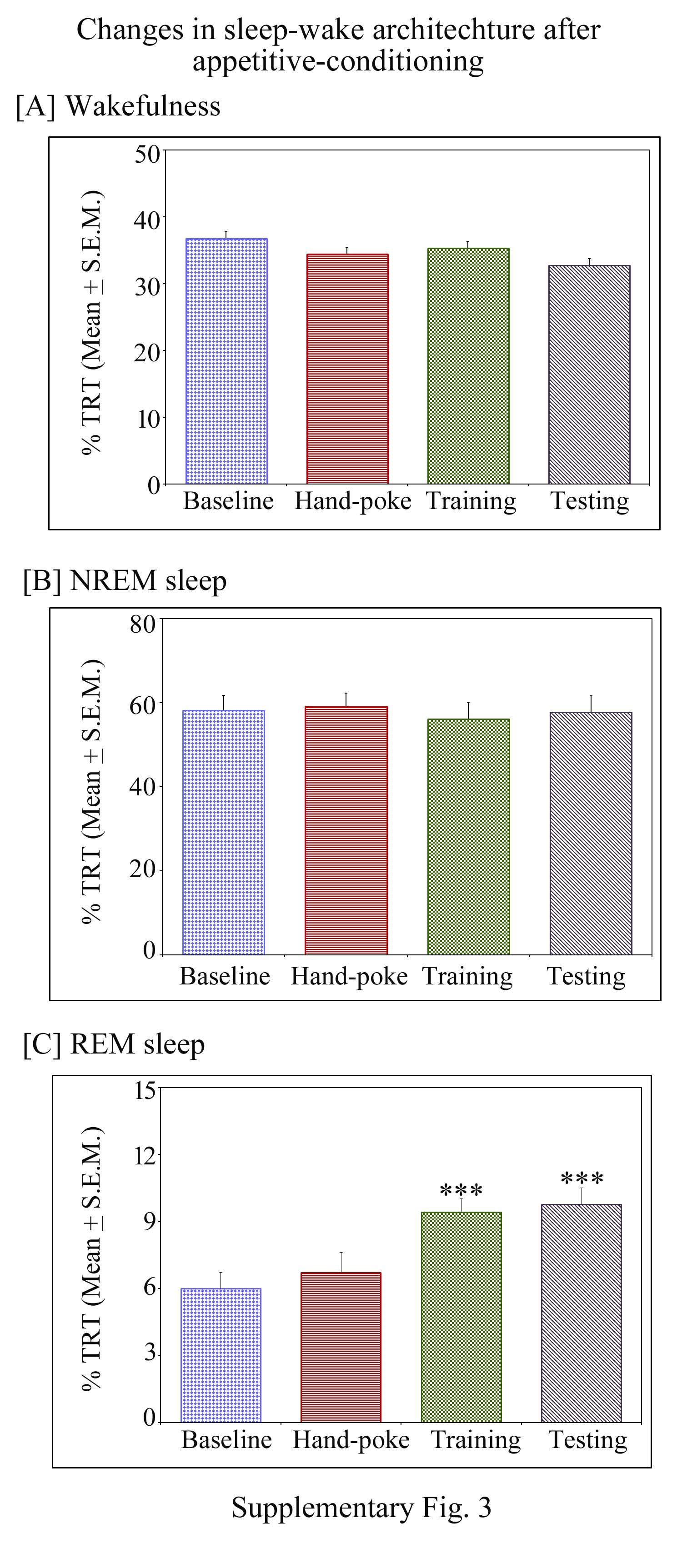

Supplement: FIGURE S3 — The percent sleep-wake amount on the baseline, hand-poke training, appetitive-conditioned training and testing days. (A) % wakefulness and (B) % NREM sleep were comparable in the baseline, hand-poke training and testing days. However, (C) % REM sleep significantly increased on appetitive-conditioned training and testing days [p < 0.001; F(3,27) = 11.17] (one-way RM ANOVA) compared to baseline day. ∗∗∗denotes Bonferroni corrected p < 0.001. [file Image_3.JPEG]
